# Amyloid fibril structure from the vascular variant of systemic AA amyloidosis

**DOI:** 10.1038/s41467-022-34636-4

**Published:** 2022-11-25

**Authors:** Sambhasan Banerjee, Julian Baur, Christoph Daniel, Peter Benedikt Pfeiffer, Manuel Hitzenberger, Lukas Kuhn, Sebastian Wiese, Johan Bijzet, Christian Haupt, Kerstin U. Amann, Martin Zacharias, Bouke P. C. Hazenberg, Gunilla T. Westermark, Matthias Schmidt, Marcus Fändrich

**Affiliations:** 1 Institute of Protein Biochemistry, Ulm University, 89081 Ulm, Germany; 2 Department of Nephropathology, Friedrich-Alexander-Universität Erlangen-Nürnberg (FAU), 91054 Erlangen, Germany; 3 Physics Department (T38), Technical University of Munich, 85748 Garching, Germany; 4 Core Unit Mass Spectrometry and Proteomics, Ulm University, 89081 Ulm, Germany; 5 Amyloidosis Center of Expertise, University Medical Center Groningen, University of Groningen, PO Box 30.001, 9700 RB Groningen, The Netherlands; 6 Department of Medical Cell Biology, Uppsala University, SE-75123 Uppsala, Sweden

**Keywords:** Protein aggregation, Cryoelectron microscopy

## Abstract

Systemic AA amyloidosis is a debilitating protein misfolding disease in humans and animals. In humans, it occurs in two variants that are called ‘vascular’ and ‘glomerular’, depending on the main amyloid deposition site in the kidneys. Using cryo electron microscopy, we here show the amyloid fibril structure underlying the vascular disease variant. Fibrils purified from the tissue of such patients are mainly left-hand twisted and contain two non-equal stacks of fibril proteins. They contrast in these properties to the fibrils from the glomerular disease variant which are right-hand twisted and consist of two structurally equal stacks of fibril proteins. Our data demonstrate that the different disease variants in systemic AA amyloidosis are associated with different fibril morphologies.

## Abstract (web summary)

This study reports the cryo-EM structures of AA amyloid fibrils from two patients with vascular AA amyloidosis. The findings imply that different disease variants in systemic amyloidosis are associated with different fibril structures.

## Introduction

Systemic AA amyloidosis is a disease in humans, wild living and captive animals^1,2^. The main amyloid deposition sites within the body are the spleen, liver and kidneys^3^. Affected patients may develop kidney symptoms, if not end-stage kidney disease^3^. Serum amyloid A1 (SAA1) is the precursor protein of AA amyloid fibrils^4^. It is an acute phase protein that becomes strongly upregulated during inflammation^5^. AA amyloid frequently occurs as a secondary complication of chronic inflammatory conditions, such as rheumatoid arthritis, tuberculosis or leprosy^6^. Retrospective analyses of historic medical specimens from patients with pulmonary tuberculosis suggested that the disease was abundant in Western countries until the first half of the 20th century^7^. However, the disease may still occur with significant numbers of cases in India and other parts of the world^8,9^.

Two variants of systemic AA amyloidosis have been described in humans^2,10^. The ‘glomerular’ disease variant is more common and characterized by extensive amyloid deposits in the glomeruli of the kidney^2^. Patients with this disease variant are typically diagnosed with proteinuria, and the extracted amyloid fibrils contain fibril proteins that produce one major band by denaturing gel electrophoresis^2,11^. This band contains SAA1 fragments that extend from residue Ser2 to mostly residues Asn64-Arg67^12^. The ‘vascular’ disease variant differs from these features by showing renal amyloid deposits that mainly affect the renal medulla and blood vessel walls rather than the glomeruli^2,10^. Patients suffering from this disease variant show only modest levels of, or no, proteinuria. Denaturing gel electrophoresis of purified fibrils reveals two types of SAA1 fragments: a smaller one from residue 2 to approximately position 44 and a larger one that extends from position 2 to approximately residue 100^2,10^.

Using cryo electron microscopy (cryo-EM), we previously determined the structure of an amyloid fibril extracted from a patient with the glomerular disease variant^13^. The fibrils in this patient belonged to essentially one fibril morphology that was structurally conserved across different patients^12,13^. The fibril showed pseudo-2_1_ screw symmetry and consisted of two structurally identical stacks of fibril proteins. The ordered core of the fibril was formed by residues Ser2-Ala55 of SAA, while the more C-terminal parts of the fibril protein were structurally disordered and/or proteolytically truncated. The fibril and the fibril’s cross-β sheets were right-handed twisted when viewed in the direction of the main fibril axis^13^. The fibrils presented significant resistance to proteolytic digestion^14^.

In this research, we determined the cryo-EM structures of amyloid fibrils that were isolated from the kidneys of two patients with the vascular variant of systemic AA amyloidosis. The two structures showed the same fibril morphology, which is significantly different from a previously described fibril from the glomerular disease variant. The differences concern the fibril symmetry, the detail fold of the fibril proteins, the involvement of two structurally different fibril proteins, the overall fibril twist, the charge distribution and the fibril’s electrostatic potential.

## Results

### Vascular AA amyloid fibrils contain two types of fibril proteins

**Fig. 1:**
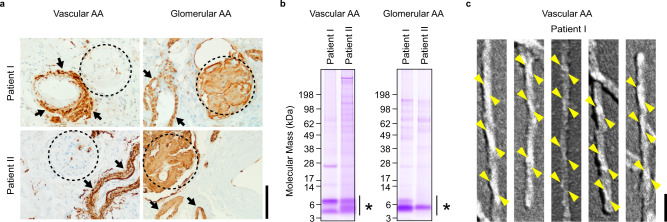
Comparison of vascular and glomerular amyloid deposits and fibrils. **a** Light microscopy images of renal tissue sections from two patients with the vascular or glomerular variant of systemic AA amyloidosis each (patients I and II). Each section was stained with anti-AA antibody (brown). Dotted lines: glomeruli. Black arrows: arteriole walls. Scale bar: 100 µm. Each micrograph is representative for at least three micrographs recorded from the respective patient. **b** Coomassie blue-stained denaturing protein gels of the fibrils extracted from patient I and II. The bars and the asterisks indicate the fibril proteins. The experiment was performed independently at least three times with similar results. **c** Scanning electron microscopy images of AA amyloid fibrils from the patient I after platinum side shadowing. Yellow (left twist) arrowheads are drawn to guide the eye. Scale bar: 50 nm. The fibril images are representative for the fibrils on at least three micrographs obtained with this sample. Source data are provided as a Source Data file.

The amyloid fibrils analyzed in this study were obtained from two patients with vascular AA amyloidosis and two patients with glomerular AA amyloidosis. The vascular patients presented up to 0.8 ± 0.3 g/24 h proteinuria and their renal amyloid deposits occurred mainly in the vessel walls (Fig. 1a, Supplementary Fig. 1). The glomeruli were degenerated and contained only low levels of amyloid (Fig. 1a, Supplementary Fig. 1). The pathology in these patients differed from patients with the glomerular disease variant, which showed a prominent glomerular involvement (Fig. 1a, Supplementary Fig. 1), and proteinuria of 5 ± 1.4 g/24 h or more. Electrophoretic analysis of amyloid fibrils purified from patients with the vascular disease pattern revealed two major fibril protein bands which migrated at ~4 and 7 kDa (Fig. 1b), whereas only one dominating fibril protein band at ~5 kDa was found with samples from patients with glomerular deposits (Fig. 1b).

Mass spectrometry previously revealed most fibril proteins from glomerular patients to extended from residue Ser2 to residues Asn64-Arg67^12^. The present analysis of fibrils from vascular patients identifies two main groups of fibrils proteins: one starting at residue Ser2 and terminating at residues Asn41-Arg47, and one starting at residue Ser2 and extending up to residues Asn64-Ala81 (Supplementary Fig. 2). All fibril proteins could be assigned to the SAA1.1 allele of SAA1 protein (Supplementary Tables 1 and 2). No other SAA family member was consistent with all observed fibril proteins, although alternative assignments were possible for some masses (Supplementary Tables 1 and 2), particularly if SAA1.1 and the other SAA family member had an identical sequence within the respective protein segment.

### Vascular AA amyloid fibrils are resistant to proteolysis and mostly left-hand twisted

Subjected to proteinase K digestion, we found vascular AA amyloid fibrils resistant to proteolysis. Approximately 80% of the fibril protein survived our proteolytic conditions for 60 min (Supplementary Fig. 3a, b). A similarly high proteolytic resistance was observed with fibrils from the glomerular disease variant (Supplementary Fig. 3c, d). By contrast, in vitro formed fibrils from human or murine SAA1 proteins become fully degraded under comparable experimental conditions as demonstrated previously^14^. The fibrils from the two vascular AA patients exhibited a width of ~9.7 nm and a pitch of ~110 nm (Supplementary Fig. 4), which is indistinguishable from glomerular AA amyloid fibrils^12^. By contrast, platinum side shadowing coupled with scanning electron microscopy revealed a mostly left-hand twist of vascular AA amyloid fibrils (Fig. 1c, Supplementary Fig. 5), which differs from the glomerular fibrils that are uniformly right-hand twisted (Supplementary Fig. 5). However, fibrils from patient I with the vascular pattern were uniformly left-hand twisted, while fibrils from patient II with the vascular pattern contained a small proportion of right-hand twisted fibrils (Fig. 1c, Supplementary Figs. 5, 6a).

### Cryo-EM structure of the vascular AA amyloid fibril

**Fig. 2:**
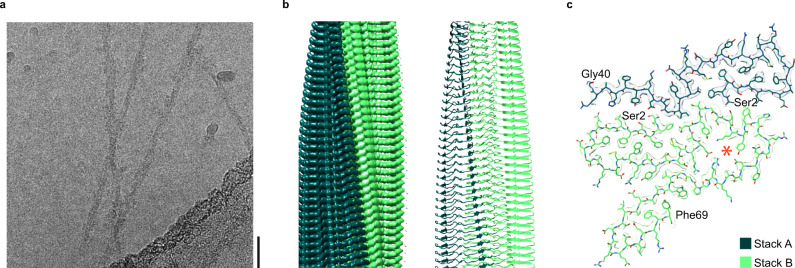
Cryo-EM structure of the vascular AA amyloid fibril. **a** Contrast-enhanced cryo-EM image of vascular AA amyloid fibrils from patient I. Scale bar: 10 nm. The micrograph is representative of 1,957 cryo-EM micrographs. **b** Side views of the reconstructed 3D map (left) and a ribbon diagram of the resulting molecular model (right). **c** Superimposition of a cross-sectional slice of the 3D map with one layer of the molecular model (stick representation). The first and the last residue of the model are indicated. Red asterisk: structural cavity. The two protein stacks corresponding to chains A and B of the fibril protein are color coded in panels **b**, **c**, as indicated in panel **c**. Source data are provided as a Source Data file.

Cryo-EM revealed a homogenous fibril ensemble for both patients (Fig. 2a, Supplementary Fig. 6b). The vast majority of fibrils visible on the micrographs appeared to belong to the same fibril morphology. This fibril morphology possessed a width of 9.4 ± 1 nm and a crossover distance of 52 ± 4 nm (*n* = 100). Reconstruction of three-dimensional (3D) maps of this fibril morphology achieved, based on the 0.143 Fourier shell correlation (FSC) criterion, a spatial resolution of 2.56 Å for the fibrils from the patient I (Supplementary Fig. 7a, b, Supplementary Table 3) and of 2.68 Å for the fibrils from patient II (Supplementary Fig. 7c, d, Supplementary Table 3). The two 3D maps were highly similar (Supplementary Fig. 7a, c), C1-symmetrical and consisted of two non-equal protein stacks (stacks A and B, Fig. 2b, c). Stack A is formed by residues Ser2 to Gly40 (Fig. 2c), while stack B contains residues Ser2 to Phe69 (Fig. 2c), reminiscent of the two groups of fibrils protein obtained by mass spectrometry (Supplementary Fig. 2). However, the protein segments in the fibril core are smaller than the total length of the fibril proteins which we determined by mass spectrometry (Fig. 2c, Supplementary Fig. 2). These data imply that the C-terminal ends of the fibril proteins are structurally disordered and thus missing in our 3D map.

### Fold of the fibril proteins in the two protein stacks

**Fig. 3:**
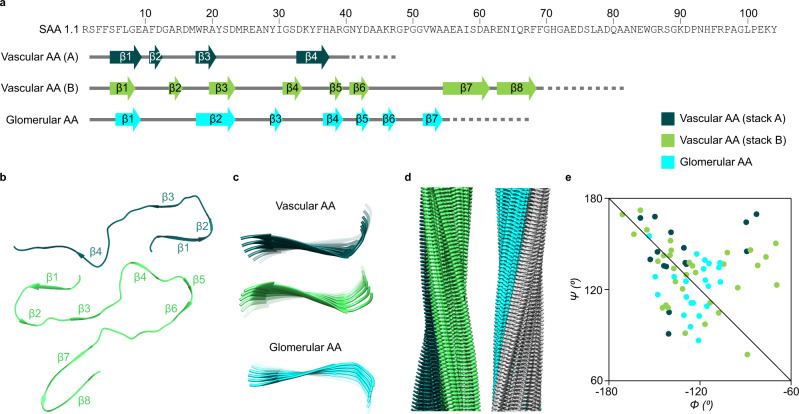
β-sheet structure in the vascular and in the glomerular fibril. **a** Sequence of SAA1.1 shown side by side with schematic representations of the fibril proteins from the vascular (stacks A and B) and glomerular fibril. Continuous gray lines: fibril core segment; dotted lines: disordered segments; arrows: β-strands. **b** Ribbon diagram of one molecular layer of the vascular fibril. β-strands are labeled with β1 to β8. **c** Ribbon diagram of residues 2–10 of the glomerular and vascular fibril (stacks A and B) showing the different twists of sheet β1. Only every third molecule along the fibril axis is shown. **d** Side views of the 3D maps of the vascular (left) and glomerular fibril (right). **e** Ramachandran plot of the residues in the β-sheets of the vascular and glomerular fibril. The data for the glomerular fibrils were plotted based on the PDB entry 6MST [https://www.rcsb.org/structure/6MST]^13^. The color coding is consistent in all panels, as explained in panel **e**. Source data are provided as a Source Data file.

The fibril proteins in both stacks show folds that are dominated by β-sheet structure (Fig. 3a) and lack the α-helical conformation of natively folded SAA1^15^. There are four cross-β sheets (β1-β4) in stack A and eight (β1-β8) in stack B (Fig. 3a, b). The β-strands are not oriented perfectly perpendicular to the main fibril axis but are slightly tilted (Fig. 3b). The β-sheets show a notable left-hand twist (Fig. 3c), which is defined here in the direction of the fibril axis. The handedness of the β-sheet twist matches the left-hand overall twist of the fibril (Fig. 3d) but differs from the right-hand twist of the fibrils and cross-β sheets in the glomerular disease variant (Fig. 3c, d). A Ramachandran plot of the β-sheet residues of the vascular fibril contains most *Ψ/Φ*-pairs on the right-hand side of the −*Φ* = *Ψ* diagonal (Fig. 3e), consistent with a left-hand β-sheet twist^16^. The glomerular fibril has the majority of the *Ψ/Φ*-pairs on the left-hand side of the diagonal (Fig. 3e), as expected for a right-hand twisted fibril. The *δ* value (*δ* = *Ψ* + *Φ*) for the β-sheet residues in stack A is 12 ± 37° and 11 ± 34° for the β-sheets of stack B. By comparison, the *δ* value was previously reported to be 8 ± 20° for the left-hand-twisted mouse AA amyloid fibril and −2 ± 20° for the right-hand twisted fibril from the glomerular variant of human AA amyloidosis^13^. The fibril proteins of stack B enclose a small and possibly water-filled cavity (Fig. 2c) that is absent in stack A. A similar cavity was previously described for the glomerular fibril^13^.

### Comparison of the fibril structural properties in vascular and glomerular AA fibrils

**Fig. 4:**
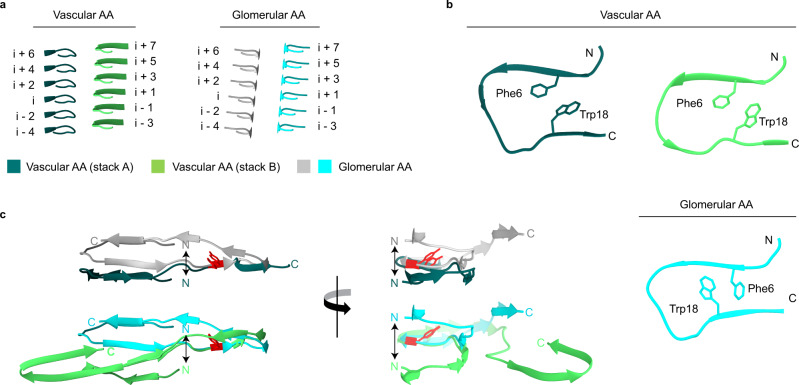
Detail structural features of the vascular and glomerular fibril. **a** Ribbon diagrams of residues Ala27-Asp33 which are close to the fibril axis, in the vascular and glomerular fibrils, showing the staggered arrangement of the two fibril protein stacks. The fibril proteins are labeled with *i* − 4 to *i* + 7. **b** Ribbon diagrams of residues Ser2-Ala20 from the N-terminal arch, indicating the flipped positions of Phe6 and Trp18. **c** Comparison of the fibril proteins in the glomerular and vascular fibrils showing the different relative positions of the fibril protein N-terminus. Top: layers *i*; bottom: layers *i* + 1. The arrow is drawn to emphasize the different positions of the fibril protein N-terminus. Tyr29 (red) serves as a reference point.

**Fig. 5:**
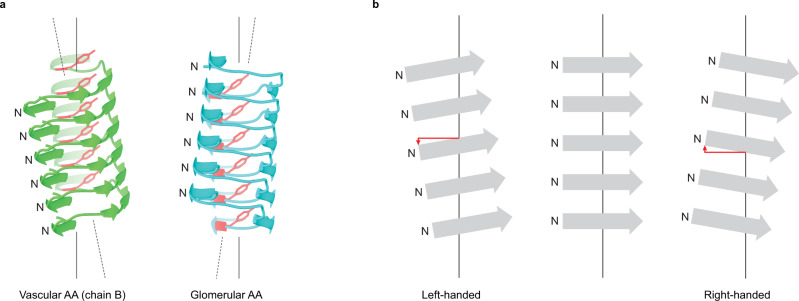
Orientation of the β-sheets relative to the fibril z-axis. **a** Different tilt of residues Ser2-Asp33 of the vascular (green, stack B) and glomerular fibril (cyan). The continuous line is parallel to the fibril *z*-axis. The dotted line is drawn to guide the eye. Tyr29 (red) serves as a reference point. **b** Schematic representation of the effect of the handedness of the fibril on the tilt of the β-strands relative to the *z*-axis. The red lines are drawn to highlight the angular tilt of the β-strands. The continuous black line indicates the fibril *z*-axis.

**Fig. 6:**
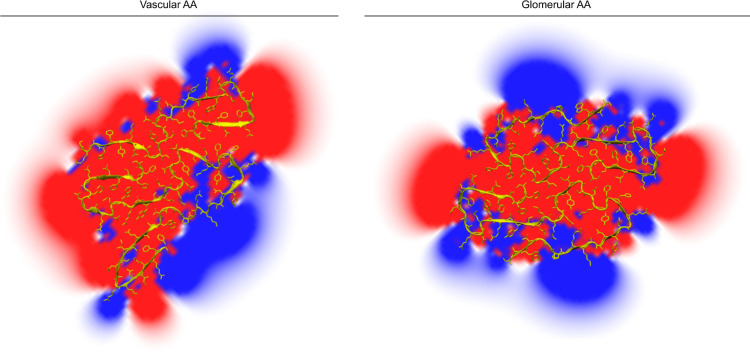
Electrostatic potential of the vascular and the glomerular fibril. The electrostatic potential of a single layer of the vascular (left) and the glomerular (right) fibril. Blue: positive charges; red: negative charges.

The vascular fibril and the previously described glomerular fibril show a staggered arrangement of their fibril protein stacks (Fig. 4a) and a similar packing of the fibril proteins in the plane of the cross-section (Supplementary Figs. 8 and 9). The fibril proteins of the two fibrils relate further by a similar β-arch structure at residues Ser2 to Ala20 (Fig. 4b). However, at least two important differences between the arches can be discerned. First, there is a revised packing of the hydrophobic core of the arch which results in a flipped orientation of residues Phe6 and Trp18 (Fig. 4b). Second, the vascular fibril involves a downwards shift of the protein N-terminus in the direction of the fibril z-axis compared with the glomerular fibril (Fig. 4c). This downwards shift is evident when comparing the z-axial position of the Cα atom of Ser2 relative to residue Tyr29. In the vascular fibril, the Cα atom of Ser2 is 3 Å lower than the Cα atom of Tyr29 in chain A and 5 Å lower in chain B. In the glomerular fibril, the Cα atom of Ser2 is 6 Å higher along the z-axis than the Cα atom of Tyr29. These very different z-axial positions of the N-terminal ends of the fibril proteins lead to the different tilt of the sheet β1 relative to the fibril z-axis (Fig. 5), which explains the different directions of the fibril twist of the glomerular and the vascular fibril (Fig. 3d). Besides these changes in the fibril’s structural anatomy, there are profound differences in the charge distribution and electrostatic potential within the fibril core. While the glomerular fibril shows a symmetrical distribution of the positive and negative charges in the fibril cross-section, the vascular fibril presents a clearly asymmetrical charge distribution (Fig. 6). There is a large basic patch on one side of the fibril cross-section and two extensive acidic patches on the other one. The net charge of one molecular layer of glomerular fibril was found to be closed to 0 at pH 7.4 while it is −2 in the vascular fibril.

## Discussion

Systemic AA amyloidosis is a worldwide occurring protein misfolding disease and one of the major forms of systemic amyloidosis^2,6^. It was previously found to occur in two human disease variants that are associated with different clinical, pathological and biochemical features^2,10^. Here we show that these two disease variants give rise to different amyloid fibril structures. The differences concern the handedness of the fibril twist and the fibril cross-β sheets (Fig. 3), the fibril symmetry (C1 or pseudo 2_1_ screw symmetry, Supplementary Fig. 8), the conformation, length and assembly of the fibril proteins (Figs. 3, 4) and the fibril’s electrostatic potential (Fig. 6). Systemic AA amyloidosis relates in these properties to other amyloid diseases where different disease variants were also found to be associated with different fibril morphologies^17–19^. However, the exact mechanism leading to these different fibril morphologies remains to be identified. Nor is it clear how the physical, chemical and morphological conditions in a patient might have resulted in these specific fibril morphologies, except that it has been suggested that fibrils with low proteolytic stability are not able to accumulate and become pathogenic in vitro^20^.

In the case of systemic AA amyloidosis, no mutational changes or other patient features could be identified that would predispose the affected individual to form the vascular or glomerular fibril structure. The different C-terminal truncation of the precursor proteins in the two disease variants might suggest an influence of the proteolytic processing of SAA1 protein in the development of the two disease variants, similar to the formation of different types of amyloid deposits by Aβ(1–40) or Aβ(1–42) peptide in the brain of Alzheimer’s disease patients^21^. However, it is not established whether proteolysis of SAA protein precedes the formation of amyloid fibrils in systemic AA amyloidosis, as would be required by such a mechanism. Furthermore, differently structured fibrils may also rather arise from different environments of fibril formation^22^ or stochastic variations in the fibril nucleation process^23^.

A particularly notable structural difference between the two types of fibrils is the handedness of their twist: left-hand in the vascular fibril and right-hand in the glomerular fibril. These differences are associated with a revised packing of the N-terminal arch (Fig. 4b) and a different z-axial position of the fibril protein N-termini relative to the central part of the fibril structure as indicated by residue Tyr29 (Fig. 5a). The different z-axial position of the N-terminus relative to the central part of the fibril leads to a different tilt of the fibril protein N-terminus (Fig. 5a), and associated with this, to a different overall twist of the β-sheets (Fig. 5b). In contrast to the exclusively left-hand twisted fibrils of the vascular patient I we noted, by platinum side shadowing, a small fraction of right-hand twisted fibrils in fibrils extracted from vascular patient II (Fig. 1c, Supplementary Figs. 5 and 6a). So far, the molecular identity of these filaments could not be identified. The cryo-EM data set could not be subdivided manually or at the 2D and 3D classification stage such that the differently twisted fibril structures observed by platinum side shadowing could be explained. Thus, it is possible that the right-hand twisted fibrils possess a very similar cross-sectional architecture to the left-hand twisted fibrils, thereby creating difficulties during 2D or 3D classification.

In addition to the differences between vascular and glomerular fibrils described above, the two fibrils show several commonalities, such as the related N-terminal arch structure (Fig. 4b), the staggered assembly of the protein stacks in the fibril (Fig. 4a), the lack of the first residue of SAA1 protein (Fig. 3a, b) and their high resistance to proteolysis (Supplementary Fig. 3). The lack of the first residue of SAA1 was previously found to be a structural prerequisite for the formation of the glomerular fibril^13^, as the burial of the fibril protein N-terminus in the densely packed core is incompatible with an additional N-terminal residue. The current vascular structure allows similar conclusions as the fibril protein N-terminus is also buried in the fibril core, indicating that the first has to be removed in order to allow the formation of the fibril structure in the vascular disease variant.

The high proteolytic resistance of the fibrils is not only observed for the two types of AA amyloid fibrils but may represent a common feature of many, if not all, ex vivo amyloid fibrils and a notable difference from most in vitro formed amyloid structures^14,20,24^. The higher proteolytic stability of ex vivo fibrils may arise from their specific morphology, as in vitro proliferation of the ex vivo fibril structure by seeding propagated the high proteolytic resistance of the seeds to the daughter filaments^25^. It was suggested that pathologically relevant fibril structures might have been selected inside the body by their ability to become highly protease stable^14,20^. Our current observation and the strong resistance of both types of AA amyloid fibrils to proteolytic digestion despite their conformational differences strongly support the view that pathologically relevant amyloid fibrils may have accumulated inside the body due to their resistance to endogenous degradation systems.

## Methods

### Ethics statement

The study complies with all relevant ethical regulations. The study is based on analyses of tissue samples from two AA patients with a vascular deposition pattern and up to four AA patients of the glomerular type. The patient characteristics are described in the Supplementary Information. Tissue materials were collected at the University Medical Center Groningen after obtaining informed consent from the patients or their relatives, who did not receive any compensation. All relevant regulations and legal requirements, including ethical approval from relevant authorities at Groningen University, were observed during material collection. The biochemical work at Ulm University was conducted based on a permission from the Ulm University Ethics Commission (203/18).

### Histological analysis of renal tissue

Frozen kidney tissue was fixed in 4% (v/v) formalin in phosphate buffered saline pH 7.4, embedded in paraffin and cut into sections of 1 µm. For detection of the amyloid deposits, sections were deparaffinized in 100% (v/v) xylene, rehydrated using a descending alcohol series and incubated for 45 min in solution I [3% (w/v) NaCl, 50% (v/v) ethanol, 0.01% (w/v) NaOH] followed by a 1.5 h incubation step in solution I supplemented with 0.5% (w/v) Congo red (Sigma Aldrich). Afterwards, sections were rinsed in distilled water, counterstained with Gill III Haematoxilin solution (Sigma Aldrich) and blued in tap water. For immunohistochemistry, sections were stained in a Ventana Benchmark stainer (Roche Diagnostics) using the following protocol. Antigen retrieval was done in cell conditioning solution 1 (Roche Diagnostics) for 54 min at 95 °C. After blocking with Avidin-Biotin block (Vector laboratories), normal goat serum (order no.: 005-000-121; lot I51472; Jackson Immuno Research Laboratories Inc.) and blotto (1:5) sections were incubated for 28 min at 37 °C in monoclonal mouse anti-AA amyloid antibody (order no.: M 0759, clone mc1, lot 20070754; DAKO; dilution 1:500). Slides were washed with 50 mM Tris pH 7.4, bound primary antibodies were detected using the “ultraView Universal DAB detection Kit” (Roche Diagnostics). Negative controls included omission of or substitution of the primary antibody with equivalent concentrations of an irrelevant murine monoclonal antibody. Photomicrographs were taken with an upright BX60 light microscope equipped with an XC30 camera at magnifications of 100–200× using cellSens imaging software (all from Olympus, Germany).

### Fibril extraction from renal tissue

A 250 mg piece of renal tissue was diced finely with a scalpel, suspended in 0.5 mL tris hydroxymethyl aminomethane (Tris) calcium buffer [20 mM Tris, 138 mM NaCl, 2 mM CaCl_2_, 0.1% (w/v) NaN_3_, pH 8.0], homogenized with ten pulses with a Kimble pellet pestle (Sigma-Aldrich) and centrifuged at 3100 × *g* for 5 min at 4 °C. The supernatant was removed and the suspension/homogenization/centrifugation cycle was repeated five more times. The final pellet was resuspended in 1 mL freshly prepared collagenase/protease inhibitor solution, which was prepared by dissolving one tablet cOmplete^TM^ ethylenediamine-tetraacetic acid (EDTA)-free protease inhibitor (Roche Diagnostics) in 7 mL Tris calcium buffer, containing 5 mg/mL crude collagenase from *Clostridium histolyticum* (Sigma-Aldrich). The mixture was incubated overnight at 37 °C under constant agitation at 700 rpm in an IKA MTS 2/4 digital table shaker, followed by the centrifugation of the incubated sample at 3100 × *g* for 30 min at 4 °C. The pellet was resuspended in 0.5 mL Tris EDTA buffer [20 mM Tris, 140 mM NaCl, 10 mM EDTA, 0.1% (w/v) NaN_3_, pH 8.0], homogenized with ten pulses of a Kimble pellet pestle (Sigma-Aldrich) and centrifuged for 5 min at 3100 × *g* and 4 °C. The supernatant was removed and the resuspension/homogenization/centrifugation cycle was repeated seven more times. The pellet was resuspended in 0.5 mL of ice-cold water and centrifuged for 5 min at 3100 × *g* in 4 °C. This step was repeated seven more times. The fibril containing supernatants from each step were retained and analyzed.

### Denaturing gel electrophoresis

The extracted fibril sample was mixed with 4× NuPAGE LDS Sample Buffer (Thermo Fischer Scientific) at a 3:1 ratio and heated at 95 °C for 10 min before it was loaded onto a 4–12% NuPAGE Bis-Tris gel (Thermo Fisher Scientific) with See Blue Plus 2 Pre-stained protein standard (Thermo Fisher Scientific) as the molecular size marker. Electrophoresis was performed with NuPAGE MES SDS Running Buffer (Thermo Fisher Scientific). After electrophoresis, the gel was stained with Coomassie solution [2.5% (w/v) Coomassie Brilliant Blue R250, 30% (v/v) ethanol and 10% (v/v) acetic acid] for 1 h at room temperature and de-stained with a solution containing 20% (v/v) ethanol and 10% (v/v) acetic acid.

### Platinum side shadowing

3.5 µL of fibril sample was added to glow discharged formvar/carbon coated 200 mesh copper grids (Electron Microscopy Sciences) and allowed to incubate for 1 min at room temperature. The excessive solvent was blotted with filter paper (Whatman) and washed three times with water and dried at room temperature for 30 min. A 1 nm thick layer of platinum on the grid was laid by evaporation at an angle of 30° with Balzers TKR 010. The grids were analyzed using a Hitachi S-5200 scanning electron microscope at an accelerating voltage of 10 kV.

### Cryo-EM

A 3.5 µL aliquot of the fibril sample was applied onto C-Flat holey carbon grids (CF – 1.2/1.3 – 4Cu, Electron Microscopy Sciences) that were glow discharged for 40 s at 20 mA using a PELCO easiGLOW glow discharge cleaning system (Ted Pella). After incubation of the sample on the grid for 30 s, the grid was double side blotted for 8 s with filter paper and plunge-frozen in liquid ethane using a Vitrobot Mark 3 (Thermo Fisher Scientific). The plunged grids were screened using a JEM-2100F transmission electron microscope (JEOL) that was operated at an accelerating voltage of 200 kV and equipped with a CMOS Camera (TVIPS). The images for the reconstruction of the 3D map were recorded using a K2-Summit detector (Gatan) on a Titan Krios transmission electron microscope (Thermo Fisher Scientific) that was operated at an accelerating voltage of 300 kV. Data acquisition parameters are listed in Supplementary Table 2. Images of 100 fibrils were used to quantify fibril width and crossover distance using Fiji^26^. Contrast enhancement was performed by using Adobe Photoshop CS6.

### Helical reconstruction

The raw data files from the microscope were converted from “tiff” to “mrcs” files and gain corrected using IMOD software^27^. The multi-frame movies were motion-corrected and averaged by using Motioncor2^28^. The contrast transfer function of the aligned and corrected micrographs was estimated by using CTFFind 4.1^29^. The helical reconstruction of the 3D map was executed in RELION 3.0 and 3.1^30^. Fibrils were manually picked on the micrographs and segments were extracted with a box size of 256 pixels and an inter-box distance of ~5% of the box length. Reference-free two-dimensional classification was executed using a regularization parameter (T) of 2. 137,936 particles were used to generate an initial 3D model using the stochastic gradient descent algorithm in RELION. The initial model was low-pass filtered to 60 Å and used to perform a reference-based 3D classification of the data with *T* = 4. The best class from the 3D classification was used to tune the resolution by changing the *T* value from 4 to 60 in a single class 3D classification. The tuned data was subjected to reference-based 3D auto-refinement of RELION. All 3D classifications and auto refinements were carried out using a central part of 30% of the intermediate asymmetrical reconstruction. To enhance the resolution of the reconstructed density map, the beam tilt, aberrations, defocus per particle and astigmatism per micrograph were adjusted by the contrast transfer function refinement tool of RELION. The data was polished with the Bayesian algorithm of RELION and the final post-processed map with a soft-edge mask was estimated with a map sharpening B-factor of −34.86 Å^2^. The reconstruction statistics are listed in Supplementary Table 3.

### Model building

The 3D density map of the fibril from patient I with a vascular deposition pattern was used to model the fibril protein conformation in COOT^31^. The glomerular type AA amyloid fibril structure^13^ was used as the starting model. The model was refined in COOT^31^ and PHENIX^32^ with Ramachandran and rotamer restraints. The validation tool in PHENIX was used to validate the atomic coordinates in terms of clash scores, rotamer outliers, Ramachandran outliers and model geometry. First, the atomic coordinates were adjusted in one layer of the fibril density map containing two polypeptide chains. After a satisfactory alignment of the main chain and the side chains to the density map, a fibril stack consisting of 6 layers, which contained twelve polypeptide chains, was generated using the pdbsymm tool of Situs^33^. The process of refinement and model-building was executed several times until a satisfactory correspondence was reached between the model and the 3D map. The statistical parameter for refinement and model building are summarized in Supplementary Table 4.

### Analysis of the electrostatic potential

The Poisson Boltzmann electrostatics (PBE) was computed for each non-terminal molecular layer of the vascular and glomerular AA fibril. The protonation states and the partial charges were assigned to a stack of ten molecular layers of each fibril at pH 7.4 through the PDB2PQR server^34^ (https://server.poissonboltzmann.org/) using PARameters for Solvation Energy forcefield^35^. The PBE for the assigned molecular layers was calculated using APBS 1.4.1^36^ via the APBS plugin provided with VMD 1.9.3^37^. All calculations were performed using standard settings as provided with VMD 1.9.3. The permittivity of the solute (protein) and implicit solvent (water) was set to 1.0 and 78.54, respectively, and a 150 mM solution of single valent ions was assumed to compute the PBE. All images depicting electrostatics have been rendered with VMD 1.9.3.

### Image representation

UCSF Chimera was used to visualize and create images for the density maps and molecular models.

### Sample statistics

Wherever appropriate, mean values are presented. Errors or error bars represent the standard deviation (SD).

### Reporting summary

Further information on research design is available in the Nature Portfolio Reporting Summary linked to this article.

## Supplementary information

Supplementary Information

Reporting Summary

## Data Availability

The cryo-EM map is deposited with the accession code EMD-14771 in the Electron Microscopy Data Bank and the fitted atomic model coordinates can be accessed from Protein Data Bank entry 7ZKY. Structural representations of the glomerular AA amyloid fibril in Figs. 3, 4, 5 and 6 and Supplementary Figs. 6 and 7 are based on the Protein Data Bank entry 6MST. Source data are provided with this paper. The materials used for the findings of the study can be obtained from the corresponding author upon reasonable request. Source data are provided with this paper.

## Source data

Source data
