# Supplementary material for: Amyloid fibril structure from the vascular variant of systemic AA amyloidosis
Source: Nat Commun. 2022 Nov 25;13:7261. doi: 10.1038/s41467-022-34636-4 (PMC9700864; doi:10.1038/s41467-022-34636-4)
Supplement: Supplementary file 1 — Supplementary Information [file 41467_2022_34636_MOESM1_ESM.pdf]

Supplementary Information

Amyloid fibril structure from the vascular variant of systemic AA amyloidosis

Sambhasan Banerjee, Julian Baur, Christoph Daniel, Peter Benedikt Pfeiffer, Manuel Hitzenberger, Lukas Kuhn, Sebastian Wiese, Johan Bijzet, Christian Haupt, Kerstin U Amann, Martin Zacharias, Bouke P.C. Hazenberg, Gunilla T. Westermark, Matthias Schmidt, Marcus Fändrich

Supporting Methods

Patient characteristics

Vascular deposition pattern of AA amyloidosis: amyloid-laden renal tissue was collected at autopsy from two patients (1 male, 1 female, 57 ± 23 years) who suffered from long-term inflammatory conditions. The patients presented with an endogenous creatinine clearance level of 35 ± 29 mL/min and proteinuria of 0.8 ± 0.3 g/24 h. Neither serum M-protein nor Bence Jones proteinuria was found. At autopsy, AA amyloid preferentially occurred in and around the vascular walls of all visceral organs, including the heart. In the kidney, amyloid was found mostly in the interstitial tissue and vascular walls. Only minor amyloid deposition was found in the glomeruli.

Glomerular deposition pattern of AA amyloidosis: amyloid-laden renal tissue was collected at autopsy from four patients (3 males, 1 female, 65 ± 5 years) who suffered from long-term inflammatory conditions. The patients presented with an endogenous creatinine clearance level of 67 ± 13 mL/min and proteinuria of 5 ± 1.4 g/24 h. Neither serum M-protein nor Bence Jones proteinuria was found. At autopsy, AA amyloid was observed in the vessel walls of many visceral organs such as the liver, thyroid and spleen. Extensive amyloid deposition was found in the glomeruli of the kidneys, while only minor amyloid deposition was seen in the interstitial tissue and vascular walls. The fibrils from these four patients were already reported in an earlier study[1].

Mass spectrometry

A 100 µL aliquot of the fibril sample was lyophilized, resuspended in 6 M guanidine hydrochloride and incubated at room temperature overnight to disaggregate the fibrils. Before

mass analysis, the sample was purified by a U3000 RSLCnano (Thermo Fisher Scientific) high pressure liquid chromatography system that was online coupled to the mass spectrometer. High pressure liquid chromatography purification was accomplished by using two columns: At first, the sample was injected on a C18 µ-precolumn (0.3 mm x 5 mm; PepMap; Dionex LC Packings; Thermo Fisher Scientific) to remove salt and other polar contaminants by washing with 0.1 % (v/v) formic acid for 5 min at a flow rate of 30 µL/min. Then, the eluate was directly transferred on an Acclaim® PepMapTM analytical column (75 µm x 500 mm, 2 µm, 100 Å pore size, Thermo Fisher Scientific) which was initially equilibrated with a mixture containing 5 % solvent B [86 % (v/v) acetonitrile, 0.1 % (v/v) formic acid] and 95 % (v/v) solvent A [0.1 % (v/v) formic acid]. The proteins were separated at a flow rate of 250 nL/min using a linear gradient from 5 to 40 % (v/v) of solvent B over 30 mins. Fractions from the Acclaim® PepMapTM analytical column (Thermo Fisher Scientific) were directly eluted into the ionization module and were further analyzed by mass spectrometry.

Samples were measured using an LTQ Orbitrap Elite system (Thermo Fisher Scientific). The mass spectrometer was equipped with a nanoelectrospray ion source and distal coated SilicaTips (FS360-20-10-D, New Objective). The instrument was externally calibrated using standard compounds (LTQ Velos ESI Positive Ion Calibration Solution, Thermo Fisher Scientific). The system was operated using the following parameters: spray voltage, 1.5 kV; capillary temperature, 250 °C; S-Lens radio frequency level, 68.9 %. The software XCalibur 2.2 SP1.48 (Thermo Fisher Scientific) was used for data-dependent tandem mass spectrometry analysis. Full scans ranging from mass to charge ratio (m/z) 370 to 1,700 were acquired in the Orbitrap at a resolution of 30,000 (at m/z 400) with automatic gain control enabled and set to 10^6 ions and a maximum fill time of 500 ms. The raw data were deconvoluted by the MASH Explorer[2] using default settings and the "Quick Deconvolution" feature. All calculated

monoisotopic masses with a score equal to or above 94 % resulting from initial m/z peaks with 5 charges or more were considered as correct and are shown in the deconvoluted mass spectra in Supplementary Figure 2. The identified fragments were assigned to SAA1.1, SAA 1.2, SAA 1.3, SAA 2.1 and SAA 2.2 by using the software mMass[3] and allowing a maximum error of 0.1 Da.

Proteinase K digestion

The fibrils extracted from patient I and II with vascular and glomerular AA amyloid deposition patterns were considered for the proteinase K digestion. The extracted fibrils were adjusted to a concentration of 0.1 mg/mL in 100 µL 20 mM Tris, 138 mM NaCl, 2 mM $CaCl_2$, 0.1 % (w/v) NaN_3, pH 8.0. From this solution, a first aliquot (15 µL) was withdrawn to indicate time point 0 min; i.e., before adding proteinase K addition. Afterwards, 1.7 µL proteinase K solution (0.02 mg/mL in water) was added to the remaining 85 µL fibril solution and incubated at 37° C. Further aliquots (15 µL) were withdrawn after incubation of the digest for 5, 10, 30 and 60 min. Digest in the aliquots was stopped by the addition of 1.5 µL phenylmethylsulfonyl fluoride solution (200 mM in methanol) immediately after aliquot removal. The aliquots were then plunged in liquid nitrogen and thawed immediately prior to sample preparation for denaturing gel electrophoresis. The quantitative evaluation of the fibril protein band on the gel was done using the program ImageJ[4]. The fibril protein amount in the aliquot before protease addition was set to 100 %.

Negative stain transmission electron microscopy

An aliquot of the fibrils isolated from the vascular AA patients I and II were placed onto a glow-discharged formvar and carbon coated 200 mesh copper grid (Electron Microscopy Sciences). The fibril sample was incubated for 1 min at room temperature before the excess

sample was removed with a filter paper. The grid was then washed three times with water and the excess water was removed with filter paper. The grid was stained three times with 2 % (w/v) uranyl acetate and after each step, the excess of the stain was removed with a filter paper. Afterwards the grid was air dried for fifteen minutes. The stained fibrils on the grids were then observed with a JEM-1400 transmission electron microscope (JEOL) accelerated at 120 kV and equipped with a TemCam-F216 camera (TVIPS). The images were recorded at 20,000 x magnification and analyzed with Fiji[5] to measure the width and pitch of the fibrils. The pitch values represent twice the cross-over distance.

Supplementary Figures

Supplementary Figure 1

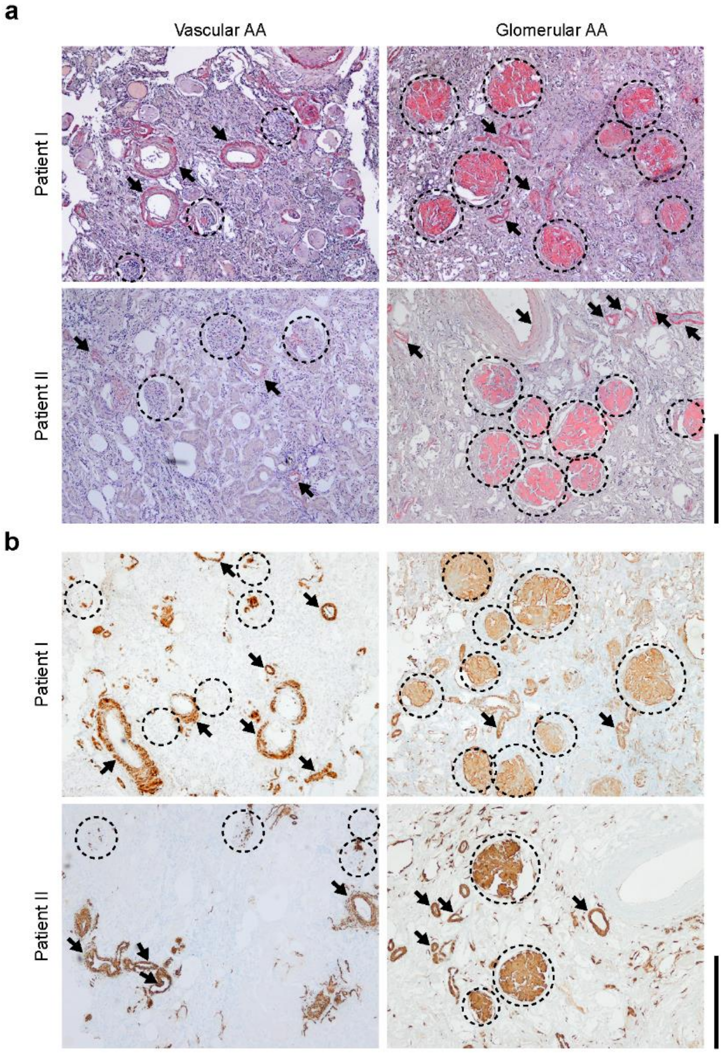

Supplementary Figure 1.

Amyloid pathology in the analyzed patients

(a) Bright field light microscopy images of Congo red-stained renal tissue sections from patients I and II with glomerular and vascular AA amyloid deposition pattern each. (b) Bright field light microscopy images of immunohistochemical stained (anti-SAA antibody) sections of renal tissue from the four patients from panel (a). Dotted lines indicate glomeruli. Arrows indicate arterioles. Scale bar: 500 µm. The micrographs are representative for at least two

micrographs recorded from each tissue section and staining protocol. Source data are provided as a Source Data file.

Supplementary Figure 2

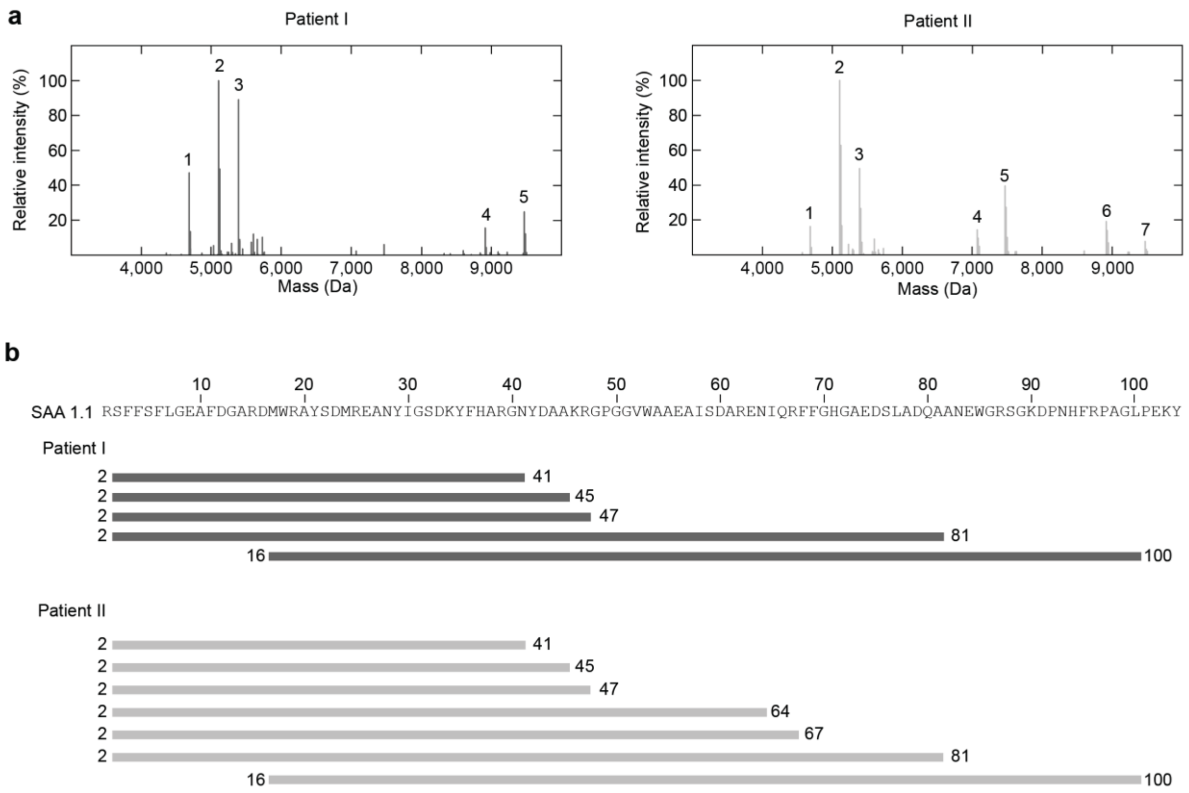

Supplementary Figure 2.

Mass spectrometric analysis of the vascular AA fibril proteins.

(a) Deconvoluted mass spectra of the extracted fibrils from patients I and II with vascular AA amyloid. See Supplementary Tables 1 and 2 for assignment of the peaks and details. (b) Graphical representation of the assigned segments of SAA1.1 protein. Source data are provided as a Source Data file.

Supplementary Figure 3

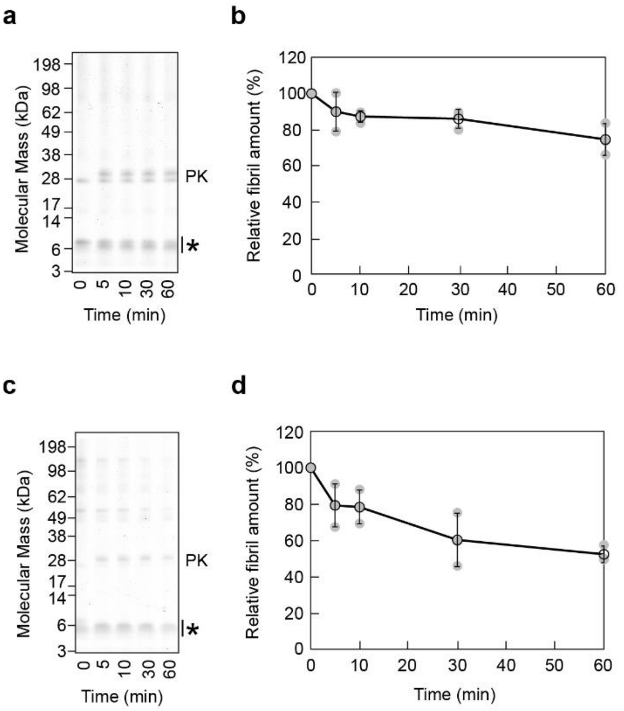

Supplementary Figure 3.

Proteolytic stability of vascular and glomerular AA amyloid fibrils.

(a) Coomassie-stained denaturing gel of vascular AA amyloid fibrils (patient I) that were incubated with proteinase K (PK) for different periods of time. Bar and asterisk indicate the fibril protein. (b) Densitometric quantification of the relative fibril protein amounts after digestion of vascular AA fibrils with proteinase K. Unfilled black symbols: average value ± SD (n = 3 independent experiments). Grey symbols: individual data points. (c) Coomassie-stained denaturing gel of glomerular AA amyloid fibrils (patient I) incubated with proteinase K (PK). (d) Densitometric quantification of the relative fibril protein amounts after digestion of glomerular AA fibrils with proteinase K. The labels in panels (c) and (d) are the same as in (a) and (b). The gel images are representative of three independent experiments processed in parallel on a single gel. Source data are provided as a Source Data file.

Supplementary Figure 4

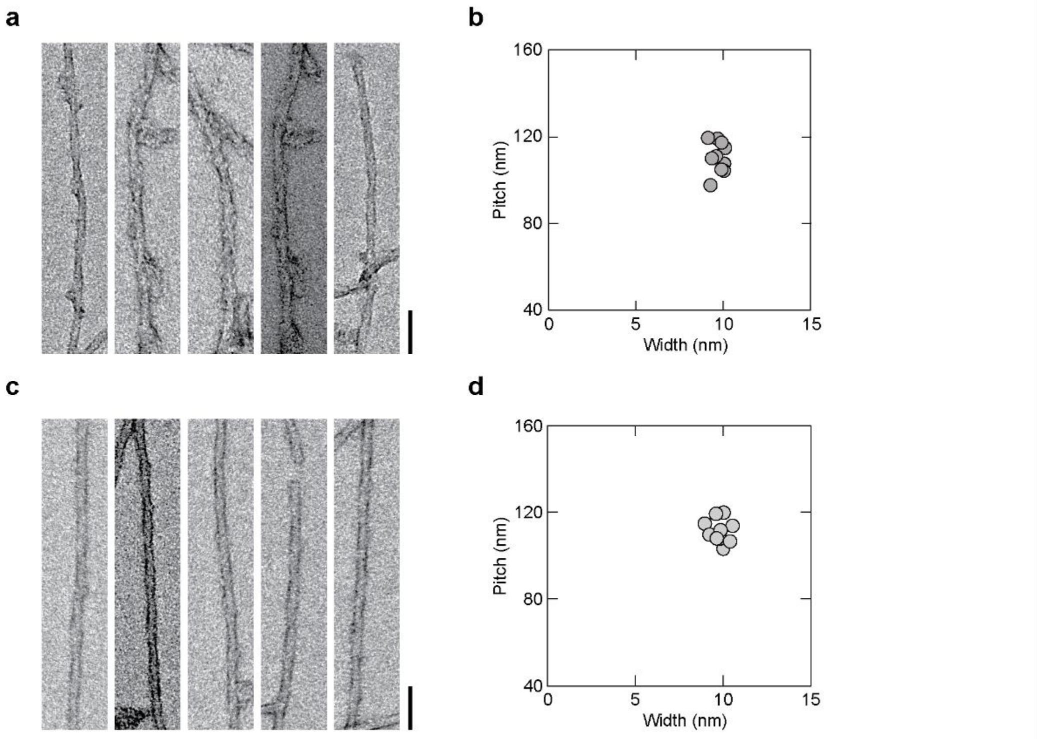

Supplementary Figure 4.

Negative-stain electron micrographs of the fibrils from the two patients with vascular amyloid deposits.

(a) Negative stain transmission electron microscopy images of the fibrils from vascular patient I. Scale bar: 50 nm. (b) Scatter plot of the pitch and width of ten representative fibrils from patient I. The width ± SD is 9.7 ± 0.3 nm, while the average pitch ± SD is 110.7 ± 6.8 nm (n = 10 individual fibrils). (c) Negative stain transmission electron microscopy images of the fibrils from patient II with vascular amyloid deposition. (d) Scatter plot of the pitch and width of ten representative fibrils from patient II. Scale bar: 50 nm. The width ± SD is 9.8 ± 0.5 nm, while the average pitch ± SD is 111.5 ± 5.2 nm (n = 10 individual fibrils). The TEM images are representative of the fibrils on at least three micrographs each. Source data are provided as a Source Data file.

Supplementary Figure 5

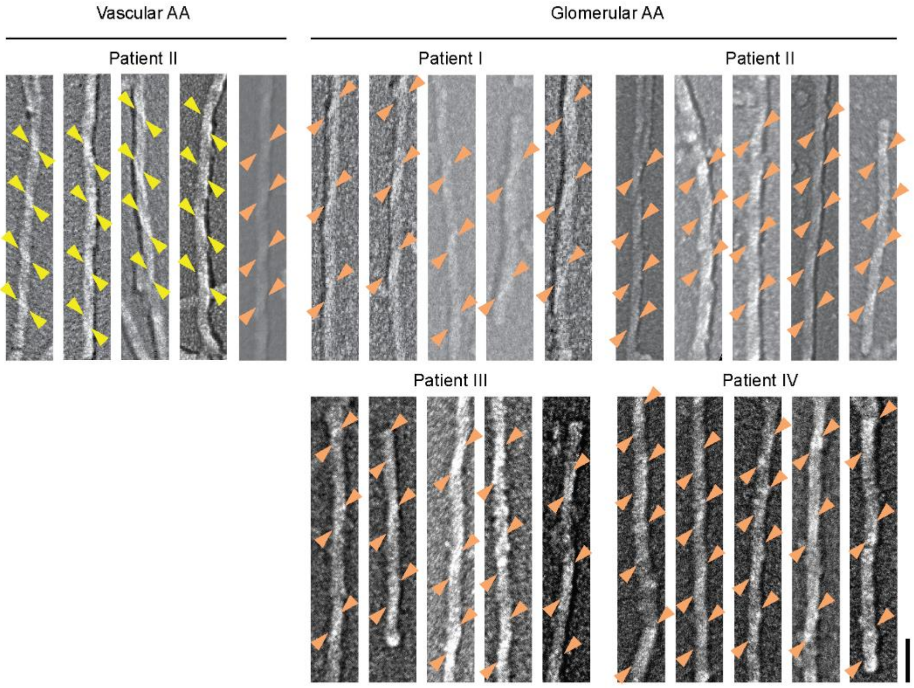

Supplementary Figure 5.

Handedness of AA fibrils from patients with a glomerular or vascular disease pattern.

Scanning electron microscopic images of the platinum side shadowed fibrils extracted from the tissues of patient II with vascular AA amyloidosis and patients I to IV of patients with glomerular AA amyloidosis. Arrowheads are drawn to guide the eye. Yellow: left-handed fibril; salmon: right-handed fibril. Scale bar: 50 nm. The images are representative for the fibrils from at least three micrographs per sample. Source data are provided as a Source Data file.

Supplementary Figure 6

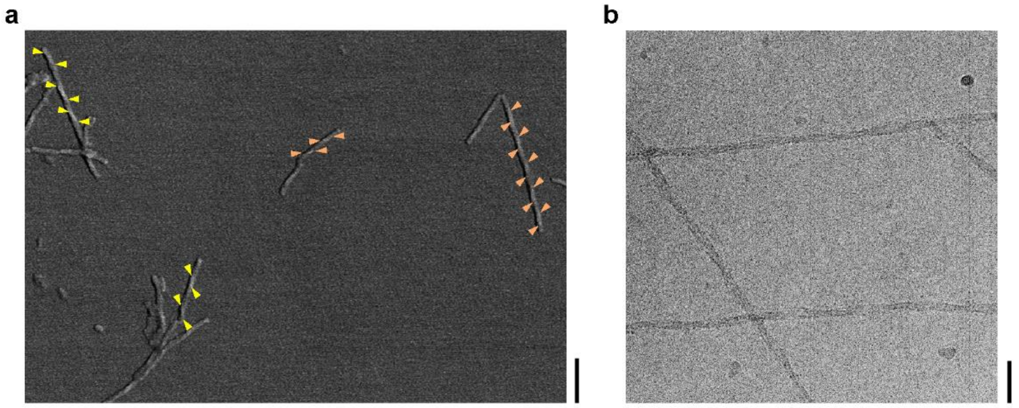

Supplementary Figure 6.

Analysis of the amyloid fibrils from patient II with vascular amyloid.

(a) Scanning electron microscopy image of platinum-side shadowed fibrils from patient II with vascular AA amyloidosis. The sample contains both left-hand (yellow arrowheads) and right-hand twisted fibrils (salmon arrowheads). Scale bar: 100 nm. The micrograph is representative of at least three micrographs obtained with this sample. (b) Contrast-enhanced cryo-EM micrograph of fibrils from this patient II. Scale bar: 10 nm. The micrograph is representative of 3,350 cryo-EM micrographs recorded with fibrils extracted from patient II. Source data are provided as a Source Data file.

Supplementary Figure 7

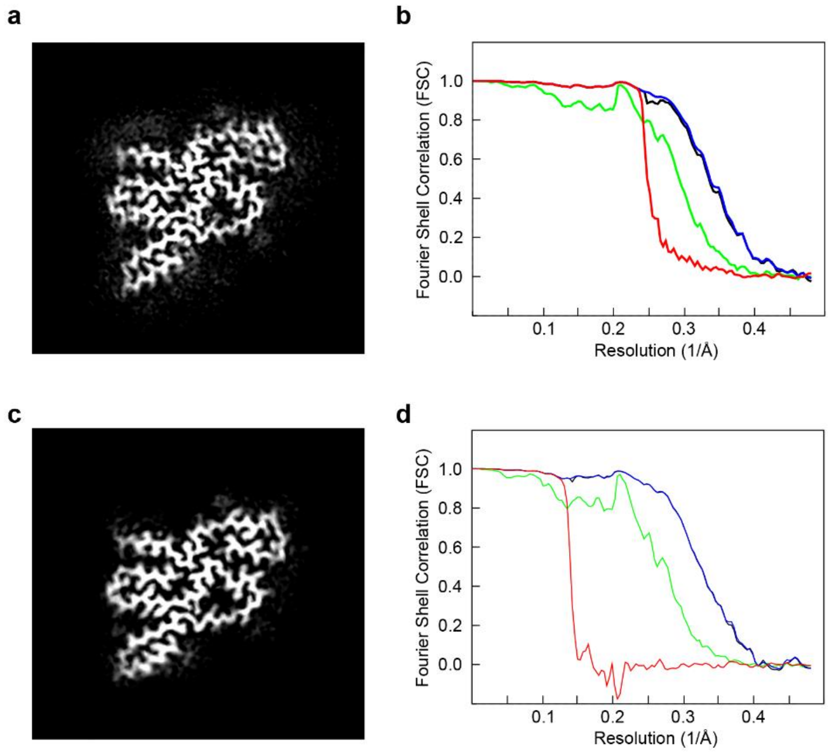

Supplementary Figure 7.

Reconstruction of an amyloid fibril morphology extracted from two patients with vascular AA amyloidosis.

(a) 5.2 Å-thick cross-sectional slice of the 3D map from vascular AA patient I. (b) FSC between the two reconstructed 3D half-maps of vascular AA patient I (c) 5.2 Å-thick cross-sectional slice of the 3D map from vascular AA patient II. (d) FSC between the two reconstructed 3D half-maps of vascular AA patient II. Black: FSC corrected; green: FSC unmasked maps; blue: FSC masked maps; red: corrected FSC phase randomized masked map. Source data are provided as a Source Data file.

Supplementary Figure 8

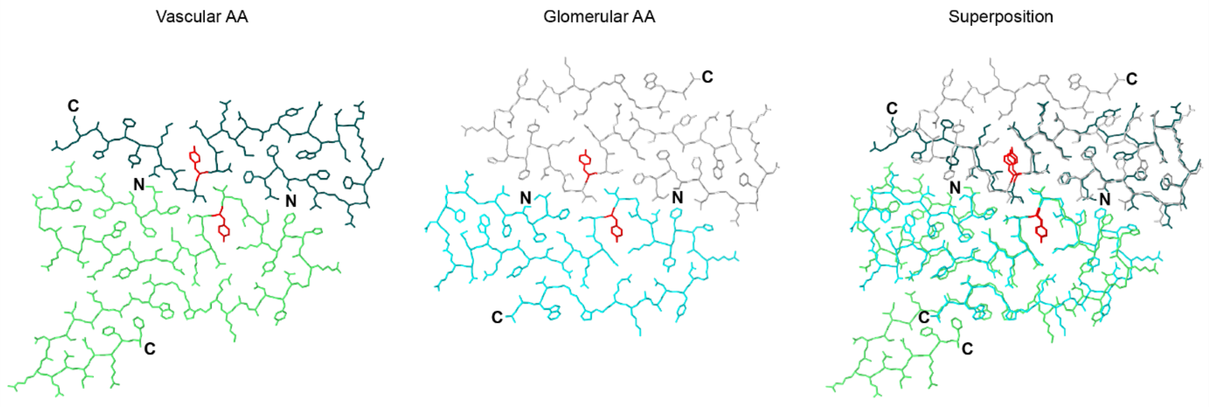

Supplementary Figure 8.

Cross-sections of the vascular and the glomerular fibril.

(a) Cross-sectional views of one molecular layer of the vascular (left) and the glomerular fibril (middle). Right image: superposition of the two cross sections. The glomerular fibril contains two equal protein stacks (cyan, grey), while the vascular fibril contains two non-equal protein stacks (light green, deep green). This figure is based on a superposition of fibril segments containing twelve molecules that were also used to assemble the panels of Fig. 4. The layers shown here refer to layers *i* and *i* + 1. Residue Tyr29 is colored red to serve as a reference point.

Supplementary Figure 9

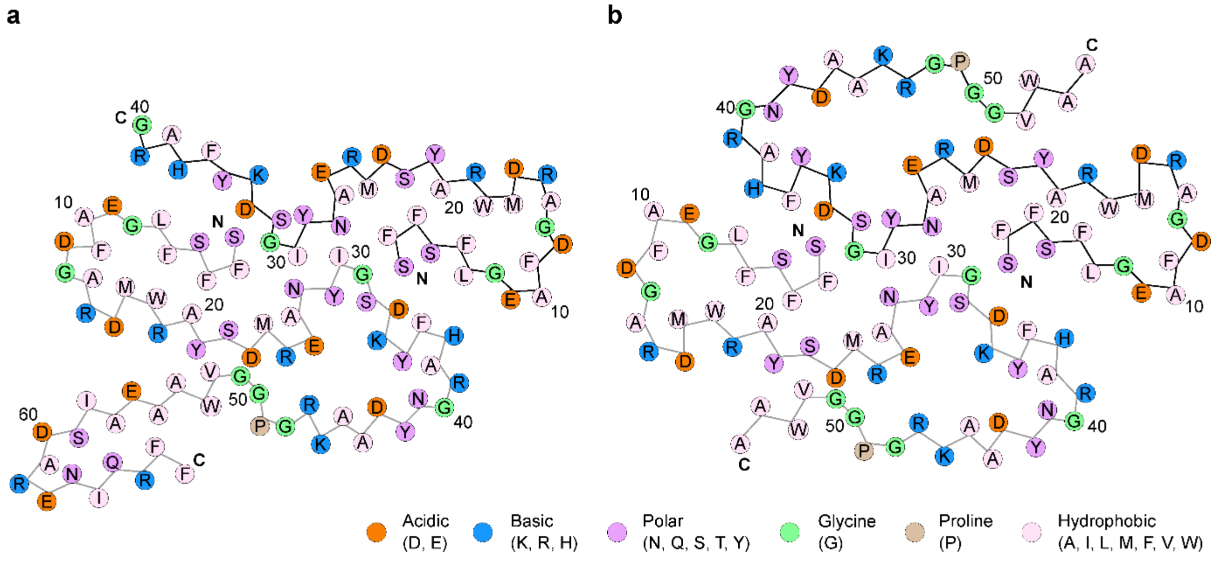

Supplementary Figure 9.

Schematic representation of the packing of the vascular and the glomerular fibril.

(a) Vascular fibril. (b) Glomerular fibril. The zig-zag lines represent the Cα-traces. Residues are color-coded according to their chemical properties, as indicated in the figure.

Supplementary Tables

Supplementary Table 1

Peak No.	Experimental Mass (Da)	Possible Assignment	Theoretical Mass (Da)
1	4,684.2	**SAA1.1(2-41)** [a,b,c,d]<br>SAA1.2(46-88)<br>SAA1.2(56-96)	4,684.1<br>4,684.2<br>4,684.2
2	5,104.3	**SAA1.1(2-45)** [a,b,c,d]<br>SAA1.2(42-88)	5,104.3<br>5,104.4
3	5,388.5	**SAA1.1(2-47)** [a,b,c,d]<br>SAA1.3(44-94)	5,388.5<br>5,388.5
4	8,912.2	**SAA1.1(2-81)**<br>SAA1.1(11-90)	8,912.1<br>8,912.1
5	9,470.4	**SAA1.1(16-100)**<br>SAA1.2(2-85)	9,470.4<br>9,470.3

Supplementary Table 1.

AA fibril proteins of vascular patient I identified by mass spectrometry.

Peak numbers refer to Supplementary Figure 2a. The table lists the experimental masses, the possible assignments and their theoretical monoisotopic masses. Only SAA1.1 is consistent with all measured masses. a: alternative assignment of that segment possible with SAA1.2; b: alternative assignment of that segment possible with SAA1.3; c: alternative assignment of that segment possible with SAA2.1; d: alternative assignment of that segment possible with SAA2.2. Bold: the simplest set of assignments.

Supplementary Table 2

Peak No.	Experimental Mass (Da)	Possible Assignment	Theoretical Mass (Da)
1	4,684.2	**SAA1.1(2-41)** [a,b,c,d]<br>SAA1.2(46-88)<br>SAA1.2 (56-96)	4,684.1<br>4,684.2<br>4,684.2
2	5,104.3	**SAA1.1(2-45)** [a,b,c,d]<br>SAA1.2(42-88)<br>SAA2.1(19-64) [d]	5,104.3<br>5,104.4<br>5,104.4
3	5,388.5	**SAA1.1(2-47)** [a,b,c,d]	5,388.5
4	7,069.3	**SAA1.1(2-64)** [a]<br>SAA1.1(24-87)<br>SAA1.2(35-98)<br>SAA1.3(37-102)<br>SAA1.3(10-72)	7,069.3<br>7,069.3<br>7,069.3<br>7,069.4<br>7,069.3
5	7,466.6	**SAA1.1(2-67)** [a]<br>SAA2.2(25-91)	7466.5<br>7466.7
6	8,912.2	**SAA1.1(2-81)**<br>SAA1.1(11-90)	8,912.1<br>8,912.1
7	9,470.4	**SAA1.1(16-100)**<br>SAA1.2(2-85)	9,470.4<br>9,470.3

Supplementary Table 2.

AA fibril proteins from vascular patient II as identified by mass spectrometry.

Peak numbers refer to Supplementary Figure 2a. The table lists the experimental masses, the possible assignments and their theoretical monoisotopic masses. Only SAA1.1 is consistent with all measured masses. a: alternative assignment of that segment possible with SAA1.2; b: alternative assignment of that segment possible with SAA1.3; c: alternative assignment of that segment possible with SAA2.1; d: alternative assignment of that segment possible with SAA2.2. Bold: the simplest set of assignments.

Supplementary Table 3

	**Vascular AA I**	**Vascular AA II**
**Microscope**	Titan Krios	Titan Krios
**Camera**	K2 Summit	K2 Summit
**Acceleration voltage (kV)**	300	300
**Magnification**	x 130,000	x 130,000
**Defocus range (µm)**	-0.8 to -2.0	-0.8 to -2.0
**Dose rate (e^-/Å^2/s)**	4.3	4.9
**Number of movie frames**	40	40
**Exposure time (s)**	10	10
**Total electron dose (e^-/Å^2)**	42.84	49.27
**Pixel size (Å)**	1.04	1.04
**Gatan imaging filter**	20 eV	20 eV
**Mode**	Counting mode	Counting mode
**Box size (pixel)**	256	256
**Inter box distance (Å)**	13.70	13.70
**Number of extracted segments**	168,956	96,599
**Number of segments after 2D classification**	137,936	-
**Number of segments after 3D classification**	77,061	52,098
**Resolution, 0.143 FSC criterion (Å)**	2.56	2.68
**Map sharpening B-Factor (Å^2)**	-34.86	-40.00
**Helical rise (Å)**	4.75	4.75
**Helical twist (°)**	-1.63	-1.40
**Symmetry imposed**	C1	C1

Supplementary Table 3.

Structural statistics of cryo-EM data collection and image processing of the vascular AA amyloid fibrils.

Supplementary Table 4

**Initial model used**	6MST
**Model resolution, 0.143 FSC criterion (Å)**	2.6
**Model composition**	
**Non-hydrogen atoms**	5,262
**Protein residues**	648
**Ligands**	0
**RMSDs**	
**Bond length (Å)**	0.012
**Bond angle (°)**	2.022
**Validation**	
**Molprobity score**	0.97
**Clash score**	2.01
**Poor rotamers (%)**	0
**Ramachandran plot**	
**Favoured (%)**	98.08
**Allowed (%)**	1.92
**Disallowed (%)**	0
**EMRinger score**	
**z score**	7.82
**score**	3.71
**Map CC**	
**ccmask**	0.61

Supplementary Table 4.

Structural statistics of model building and refinement.

The model was built based on the 3D map of the fibril from patient I with a vascular amyloid deposition pattern.

References

1. Liberta, F. *et al.* Morphological and primary structural consistency of fibrils from different AA patients (common variant). *Amyloid* **26**, 164–170 (2019).
2. Wu, Z. *et al.* MASH Explorer: A Universal Software Environment for Top-Down Proteomics. *J Proteome Res* **19**, 3867–3876 (2020).
3. Niedermeyer, T. H. J. & Strohalm, M. mMass as a Software Tool for the Annotation of Cyclic Peptide Tandem Mass Spectra. *PLoS One* **7**, e44913 (2012).
4. Schneider, C. A., Rasband, W. S. & Eliceiri, K. W. NIH Image to ImageJ: 25 years of image analysis. *Nat Methods* **9**, 671–675 (2012).
5. Schindelin, J. *et al.* Fiji: An open-source platform for biological-image analysis. *Nature Methods* vol. 9 676–682 (2012).
