# Supplementary material for: Amyloid fibril structure from the vascular variant of systemic AA amyloidosis
Source: Nat Commun. 2022 Nov 25;13:7261. doi: 10.1038/s41467-022-34636-4 (PMC9700864; doi:10.1038/s41467-022-34636-4)
Supplement: Supplementary file 2 — Reporting Summary [file 41467_2022_34636_MOESM2_ESM.pdf]

nature portfolio

Corresponding author(s): Prof. Dr. Marcus Fändrich

Last updated by author(s): Oct 14, 2022

Reporting Summary

Nature Portfolio wishes to improve the reproducibility of the work that we publish. This form provides structure for consistency and transparency in reporting. For further information on Nature Portfolio policies, see our Editorial Policies and the Editorial Policy Checklist.

Statistics

For all statistical analyses, confirm that the following items are present in the figure legend, table legend, main text, or Methods section.

n/a	Confirmed	
☐	☒	The exact sample size (*n*) for each experimental group/condition, given as a discrete number and unit of measurement
☐	☒	A statement on whether measurements were taken from distinct samples or whether the same sample was measured repeatedly
☒	☐	The statistical test(s) used AND whether they are one- or two-sided<br>*Only common tests should be described solely by name; describe more complex techniques in the Methods section.*
☒	☐	A description of all covariates tested
☒	☐	A description of any assumptions or corrections, such as tests of normality and adjustment for multiple comparisons
☐	☒	A full description of the statistical parameters including central tendency (e.g. means) or other basic estimates (e.g. regression coefficient) AND variation (e.g. standard deviation) or associated estimates of uncertainty (e.g. confidence intervals)
☒	☐	For null hypothesis testing, the test statistic (e.g. *F*, *t*, *r*) with confidence intervals, effect sizes, degrees of freedom and *P* value noted<br>*Give P values as exact values whenever suitable.*
☒	☐	For Bayesian analysis, information on the choice of priors and Markov chain Monte Carlo settings
☒	☐	For hierarchical and complex designs, identification of the appropriate level for tests and full reporting of outcomes
☒	☐	Estimates of effect sizes (e.g. Cohen's *d*, Pearson's *r*), indicating how they were calculated

Our web collection on statistics for biologists contains articles on many of the points above.

Software and code

Policy information about availability of computer code

Data collection	SerialEM v3.6.0beta, cellSens imaging software
Data analysis	Relion v3.0, 3.1, Motioncor2, CTFfind 4.1, IMOD v4.9.4, Coot v0.9.1, PHENIX v1.2, ImageJ v1.52p, UCSF Chimera v1.15, Situs, PDB2PQR server, VMD 1.9.3, XCalibur 2.2 sp1.48, mMAss v5.5.0

For manuscripts utilizing custom algorithms or software that are central to the research but not yet described in published literature, software must be made available to editors and reviewers. We strongly encourage code deposition in a community repository (e.g. GitHub). See the Nature Portfolio guidelines for submitting code & software for further information.

Data

Policy information about availability of data

All manuscripts must include a data availability statement. This statement should provide the following information, where applicable:

- Accession codes, unique identifiers, or web links for publicly available datasets
- A description of any restrictions on data availability
- For clinical datasets or third party data, please ensure that the statement adheres to our policy

The cryo-EM map is deposited with the accession code EMD-14771 [https://www.ebi.ac.uk/pdbe/entry/emdb/EMD-14771] in the Electron Microscopy Data Bank and the fitted atomic model coordinates can be accessed from Protein Data Bank entry 7ZKY [https://doi.org/10.2210/pdb7ZKY/pdb]. Structural representations of the glomerular AA amyloid fibril in Figures 3, 4, 5 and 6 and Supplementary Figures 6 and 7 are based on the Protein Data Bank entry 6MST [https://www.rcsb.org/structure/6MST]. Source data are provided with this paper. The materials used for the findings of the study can be obtained from the corresponding author upon

reasonable request.

Human research participants

Policy information about studies involving human research participants and Sex and Gender in Research.

Reporting on sex and gender	The patient details are presented as a cohort, no direct identifiers are presented in the paper.
Population characteristics	Two patients with vascular variant of AA amyloidosis (1 male and 1 female). Tissue from up to four patients with glomerular variant of AA amyloidosis (3 males and 1 female) was used to illustrate known features of the previously described disease variant.
Recruitment	The patients were selected on the pattern of renal AA amyloid deposition - vascular or glomerular deposition. The patients were not selected biased on gender, sex or any other relevant parameters that influences the study.
Ethics oversight	Tissue materials were collected at the University Medical Center Groningen after obtaining informed consent from the patients or their relatives, who did not receive any compensation. All relevant regulations and legal requirements, including ethical approval from relevant authorities at Groningen University, were observed during material collection. The biochemical work at Ulm University was conducted based on a permission from the Ulm University Ethics Commission (203/18).

Note that full information on the approval of the study protocol must also be provided in the manuscript.

Field-specific reporting

Please select the one below that is the best fit for your research. If you are not sure, read the appropriate sections before making your selection.

- [x] Life sciences
- [ ] Behavioural & social sciences
- [ ] Ecological, evolutionary & environmental sciences

For a reference copy of the document with all sections, see nature.com/documents/nr-reporting-summary-flat.pdf

Life sciences study design

All studies must disclose on these points even when the disclosure is negative.

Sample size	No established methods or statistical calculations were considered for the sample size. The samples size is dependent on the availability of the tissue. The glomerular AA amyloidosis is more common and has four cases, while the vascular AA amyloidosis is rarer and has two cases.
Data exclusions	Vascular AA patient I: 77,061 helical segments were retained from initially 168,956 helical segments.<br>Vascular AA patient II: 52,098 helical segments were retained from initially 96,599 helical segments.
Replication	The reconstructed density map confirmed by two consecutive dataset from two different patients. The reproducibility of other experimental results relies on atleast three independent replicated experiments, unless stated otherwise.
Randomization	Not relevant to the study as it is a two case study from one cohort.
Blinding	The analysis is a two case study that does not report statistical comparisons between different cohorts.

Reporting for specific materials, systems and methods

We require information from authors about some types of materials, experimental systems and methods used in many studies. Here, indicate whether each material, system or method listed is relevant to your study. If you are not sure if a list item applies to your research, read the appropriate section before selecting a response.

Materials & experimental systems

n/a	Involved in the study	
[ ]	[x]	Antibodies
[x]	[ ]	Eukaryotic cell lines
[x]	[ ]	Palaeontology and archaeology
[x]	[ ]	Animals and other organisms
[x]	[ ]	Clinical data
[x]	[ ]	Dual use research of concern

Methods

n/a	Involved in the study	
[x]	[ ]	ChIP-seq
[x]	[ ]	Flow cytometry
[x]	[ ]	MRI-based neuroimaging

Antibodies

Antibodies used	goat serum (Order No.: 005-000-121; Lot I51472; Jackson Immuno Research Laboratories Inc.), monoclonal mouse anti-AA amyloid antibody (Order No.: M 0759, clone mc1, Lot 20070754; DAKO; dilution 1:500)

Validation

The following text was taken from the company's web site (https://www.agilent.com/cs/library/packageinsert/public/SSM0759CEEFG_01.pdf) and is reproduced here with small modifications.

Intended use
For in vitro diagnostic use.
Monoclonal Mouse Anti-Human Amyloid A, Clone mc1, is intended for use in immunocytochemistry. The antibody labels amyloid A (AA) in tissues, and aids in the identification and classification of AA-amyloidosis. Immunocytochemical staining using Dako Monoclonal Mouse Anti-Human Amyloid A in combination with Congo Red staining (1), or particularly Congo Red fluorescence (2), is much more sensitive than Congo Red staining alone. Differential identification is aided by the results from a panel of antibodies. Interpretation must be made within the context of the patient's clinical history and other diagnostic tests by a qualified pathologist.

Introduction
Amyloidosis is a group of diseases that have in common the extracellular deposition of fibrillar proteins with a specific biochemical conformation known as ß-pleated sheets. Approximately 20 different precursor proteins that may be deposited as amyloid fibrils have been identified (3). Amyloid proteins deposited in the tissues can be identified by Congo Red staining, and chemically classified via amino acid sequence studies, by immunochemistry, or via immunocytochemistry (1). Amyloid A (AA) is an extracellular deposited insoluble fibrillar protein, highly resistant to proteolytic degradation, and produced from the precursor protein, serum amyloid A (SAA) (3). Before the therapy of a suspected amyloid disease can be planned, both the presence of amyloid and its chemical origin must be known (2).
Reagent provided
Monoclonal mouse antibody provided in liquid form as cell culture supernatant dialysed against 0.05 mol/L Tris/HCL, pH 7.2, and containing 15 mmol/L NaN3. Clone: mc1 (4, 5). Isotype: IgG2a, kappa. Mouse IgG concentration: see label on vial.
Immunogen
An equal mixture of human amyloid A coupled to horseradish peroxidase and human amyloid A coupled to high molecular weight kininogen (5).

Specificity
In micro-ELISA, the antibody reacts with amyloid A and the serum precursor of amyloid A indicating cross-reactivity among amyloid A protein and the serum precursor of amyloid A. In contrast, no reactivity to other non-AA amyloid fibril proteins or human serum proteins, such as albumin, transferrin, and IgG is seen (4, 5). In immunocytochemistry, the antibody labels tissues from AA patients, but shows no reactivity with a host of unknown antigens in tissue sections of various organs (5), nor with amyloid types Akappa, Alambda and amyloid fibril proteins in familial amyloid polyneuropathy and senile cardiovascular amyloidosis, respectively. Neither are Alzheimer's amyloid plaques, amyloid in microangiopathy, lichen amyloidosis, and senile islets of Langerhans labelled by the antibody (5-7).

Precautions
1. For professional users.
2. This product contains sodium azide (NaN3), a chemical highly toxic in pure form. At product concentrations, though not classified as hazardous, sodium azide may react with lead and copper plumbing to form highly explosive build-ups of metal azides. Upon disposal, flush with large volumes of water to prevent metal azide build-up in plumbing.
3. As with any product derived from biological sources, proper handling procedures should be used.

Storage
Store at 2-8 degree C. Do not use after expiration date stamped on vial. If reagents are stored under any conditions other than those specified, the user must verify the conditions. There are no obvious signs to indicate instability of this product. Therefore, positive and negative controls should be run simultaneously with patient specimens. If unexpected staining is observed which cannot be explained by variations in laboratory procedures and a problem with the antibody is suspected, contact our Technical Services.

Specimen preparation
Paraffin sections: The antibody can be used for labelling paraffin-embedded tissue sections fixed in formalin. Pre-treatment of tissues with proteinase K or heat-induced epitope retrieval is recommended. For heat-induced epitope retrieval, optimal results are obtained with Dako Target Retrieval Solution, code No. S 1700, Dako Target Retrieval Solution, High pH, code No. S 3308, 10 mmol/L citrate buffer, pH 6.0, or 10 mmol/L Tris buffer, 1 mmol/L EDTA, pH 9.0. The tissue sections should not dry out during the treatment or during the following immunocytochemical staining procedure.
Frozen sections and cell preparations: The antibody can be used for labelling frozen sections (5).

Staining procedure
Dilution: Monoclonal Mouse Anti-Human Amyloid A, code No. M 0759, may be used at a dilution range of 1:50-1:100 when applied on formalin-fixed, paraffin-embedded sections of human kidney from a patient with amyloidosis A and using 20 minutes heat-induced epitope retrieval in Dako Target Retrieval Solution, code No. S 1700, and 30 minutes incubation at room temperature with the primary antibody. Optimal conditions may vary depending on specimen and preparation method, and should be determined by each individual laboratory. The recommended negative control is Dako Mouse IgG2a, code No. X 0943, diluted to the same mouse IgG concentration as the primary antibody. Unless the stability of the diluted antibody and negative control has been established in the actual staining procedure, it is recommended to dilute these reagents immediately before use, or dilute in Dako Antibody Diluent, code No. S 0809. Positive and negative controls should be run simultaneously with patient specimen.
Visualization: DAKO LSAB™+/HRP kit, code No. K 0679, and DAKO EnVision™+/HRP kits, code Nos. K 4004 and K 4006, are

recommended. For frozen sections and cell preparations, the Dako APAAP kit, code No. K 0670, is a good alternative if endogenous peroxidase staining is a concern. Follow the procedure enclosed with the selected visualization kit.
Automation: The antibody is well-suited for immunocytochemical staining using automated platforms, such as the Dako Autostainer.
Product-specific limitations
Cross-reactivity with the serum precursor of amyloid A has been observed (5, 6).

Performance characteristics
Amyloid A labelled by the antibody has an extracellular localization in the majority of cases

References/ Références/ Literatur

1. Linke RP, Gärtner HV, Michels H. High-sensitivity diagnosis of AA amyloidosis using Congo Red and immunohistochemistry detects missed amyloid deposits. J Histochem Cytochem 1995;43:863-9.
2. Linke RP. Highly sensitive diagnosis of amyloid and various amyloid syndromes using Congo red fluorescence. Virchows Arch 2000;436:439-48.
3. Sipe JD, Cohen AS. History of the amyloid fibril [review]. J Struct Biol 2000;130:88-98.
4. Linke RP. Identification of amyloid protein AA with a monoclonal antibody. Blut 1982;45:407-9.
5. Linke RP. Monoclonal antibodies against amyloid fibril protein AA. Production, specificity, and use for immunohistochemical localization and classification of AA-type amyloidosis. J Histochem Cytochem 1984;32:322-8.
6. Linke RP. Identification of AA-type amyloid in tissue sections using monoclonal antibodies. In:Peeters H, editor. Protides Biol Fluids. Oxford: Pergamon Press; 1983. p. 835-8.
7. Linke RP, Nathrath WBJ, Eulitz M. Classification of amyloid syndromes from tissue sections using antibodies against various amyloid fibril proteins: report of 142 cases. In: Glenner CG, Osserman EF, Benditt EP, Calkins E, Cohen AS, Zucker-Franklin D, editors. Amyloidosis. Amsterdam: Plenum Publishing Corporation; 1986. p. 599-605
